# Puerarin Attenuates White Matter Injury and Blood–Brain Barrier Disruption After Intracerebral Hemorrhagic Stroke via cGAS-STING Axis

**DOI:** 10.3390/biology15030277

**Published:** 2026-02-03

**Authors:** Yetong Ouyang, Lijia Yu, Yue Shi, Zhilin Chen, Xiaohui Tang, Jiayi Jin, Zhexue Huang, Xiaoshun Tang, Bing Zhu, Xijin Wang

**Affiliations:** 1 Center for Translational Neurodegeneration and Regenerative Therapy, School of Medicine, Tongji University, Shanghai 200333, China; 2 Clinical Center for Brain and Spinal Cord Research, Tongji University, Shanghai 200333, China

**Keywords:** Puerarin, cGAS-STING pathway, intracerebral hemorrhage (ICH), white matter injury (WMI), blood–brain barrier (BBB), network pharmacology

## Abstract

White matter injury (WMI) and blood–brain barrier (BBB) disruption contribute to neurological and cognitive deficits in intracerebral hemorrhage (ICH), with no effective pharmacological treatments available. Puerarin, with anti-inflammatory, anti-apoptotic, and antioxidant properties, exhibits neuroprotective potential. Here, mice subjected to ICH were treated with puerarin for 14 days. Neurological function, cerebral perfusion, and BBB integrity were assessed using behavioral tests, laser speckle imaging, Evans blue assays, immunofluorescence, Western blotting, and MRI. Integrated transcriptomics, machine learning, network pharmacology, molecular docking, and dynamics simulations were used to identify key targets. Puerarin improved neurological outcomes, reduced BBB permeability, enhanced microvascular perfusion, and attenuated WMI. Twenty-six hub genes were identified, with *PARP1* and *AKT1* correlated with *OLIG2* and *MBP*, enriched in the cGAS-STING and AKT1-mTOR pathways. Molecular simulations indicated stable puerarin–cGAS interactions, validated experimentally: puerarin suppressed cGAS-STING activation, reduced oligodendrocyte apoptosis, and promoted remyelination. These results provide new insights into ICH pathogenesis and support puerarin as a potential therapeutic agent for BBB disruption and WMI.

## Simple Summary

Intracerebral hemorrhage (ICH) is the most fatal subtype of stroke, often leading to severe white matter injury (WMI) and blood–brain barrier (BBB) disruption, which culminate in devastating neurological deficits. Currently, no effective pharmacological treatments exist. In this study, we investigated the protective effects of puerarin on WMI and BBB integrity in ICH. Using a combination of network pharmacology, transcriptomic analysis, and machine learning, we identified key genes associated with WMI in ICH. Molecular docking and molecular dynamics simulations were then employed to evaluate the stability of puerarin potential binding to these target proteins. Importantly, our study demonstrates for the first time that puerarin can modulate the cGAS-STING signaling pathway in a mouse model of ICH, thereby alleviating white matter damage and BBB disruption.

## 1. Introduction

Intracerebral hemorrhage (ICH) is the most fatal subtype of stroke, representing 10–15% of all stroke cases while accounting for more than 40% of stroke-related deaths [1]. With an estimated 2–5 million cases annually worldwide, intracerebral hemorrhage (ICH) poses a substantial public health burden. Despite advances in surgical management, including minimally invasive thrombectomy, severe white matter injury (WMI) and blood–brain barrier (BBB) disruption frequently occur, resulting in devastating neurological outcomes [2], such as persistent deficits, cognitive impairment, and loss of consciousness or coma. The prognosis remains extremely poor, with 30-day mortality exceeding 40%, approximately half of which occurs within the first 48 h [3]. Following ICH, myelin repair is primarily mediated by the differentiation of neural precursor cells into mature oligodendrocytes, a process that is significantly regulated by neuroinflammation. Mechanical pressure from hemoglobin deposition and cerebral edema following ICH damages white matter and compromises its integrity, a process further exacerbated by additional pathological factors, including tissue swelling, elevated reactive oxygen species (ROS), and deleterious blood-derived components such as ferrous ions and thrombin [4]. M1-polarized microglia further amplify injury by generating ROS, secreting inflammatory cytokines, and releasing complement C3 [5]. These cascades collectively contribute to fiber tract degeneration, oligodendrocyte depletion, and myelin loss. In our previous study, we identified pathogenic KARS variants (p. R477H, p. P505S) associated with progressive cognitive impairment and leukoencephalopathy and indicated important roles of KARS in the regulation of oligodendrocyte differentiation and myelination during brain development [6]. Previous single-cell and spatial transcriptomic studies have suggested that Transcriptomic studies have indicated that oligodendrocyte loss significantly contributes to early white matter damage following ICH, with this process occurring as early as one hour post-hemorrhage and peaking at 24 h [7]. Consequently, preventing oligodendrocyte death and promoting myelin repair constitute critical strategies for neurological functional recovery. Several emerging studies have indicated that transplantation of human oligodendrocyte precursor cells (OPCs) or early hematoma evacuation may attenuate white matter injury following neonatal intracerebral hemorrhage; however, highly effective non-invasive pharmacological interventions remain unavailable [8]. Therefore, identifying novel therapeutic strategies to mitigate WMI and BBB disruption while promoting functional recovery represents an urgent clinical need.

Puerarin, a bioactive compound extracted from Pueraria lobata, has been traditionally employed in Chinese medicine for the treatment of cardiovascular and cerebrovascular disorders [9]. Emerging evidence indicates its therapeutic potential across multiple neuropathological conditions, including traumatic brain injury and neurodegenerative diseases [10]. These neuroprotective effects are partly mediated by the inhibition of apoptosis and the enhancement of endogenous clearance mechanisms. Mechanistically, puerarin regulates cellular processes by promoting anti-apoptotic Bcl-2/Bax signaling, activating the Sirt3/SOD2 antioxidant axis, stimulating the PI3K/AKT1 pathway, and inhibiting NF-κB signaling [11]. Previous investigations have suggested that puerarin promotes white matter repair in stroke by facilitating microglial clearance of damaged myelin debris [12]. Furthermore, another study suggested that puerarin may modulate metabolic pathways via the brain–gut axis, potentially influencing myelin lipid metabolism and synthesis in a manner analogous to its effects on systemic lipid absorption [13]. Nevertheless, the specific mechanisms by which puerarin ameliorates WMI and BBB disruption following ICH remain poorly understood [14]. However, a specific knowledge gap remains regarding puerarin’s effects on neuronal survival, fiber tract integrity, and myelin preservation at lesion sites [15]. It is still unclear whether puerarin can promote the repair of white matter and the blood–brain barrier following hemorrhagic injury, representing a critical gap in ICH therapeutics.

ICH triggers a sequential pathogenic cascade wherein the initial hematoma elicits persistent neuroinflammation and oxidative stress, potentially contributing to oligodendrocyte death. Consequently, WMI and exacerbated BBB disruption ensued, ultimately culminating in long-term neurological deficits. Therefore, targeting such inflammation represents a rational therapeutic strategy to preserve white matter integrity and improve recovery. In this context, the natural isoflavone puerarin has shown promise for its potential to interrupt this cascade, particularly through modulation of the cGAS–STING pathway—a central mediator of sterile inflammation. By potentially inhibiting cGAS–STING activation, puerarin may attenuate downstream neuroinflammatory and oxidative stress responses, thereby protecting oligodendrocytes, mitigating WMI and BBB disruption, and ultimately reducing neurological deficits following ICH. In this study, we examined how puerarin reduces WMI and decreases BBB permeability in ICH. We used network pharmacology, transcriptomic analysis, and machine learning to identify key genes related to WMI in ICH. We then performed molecular docking and molecular dynamics simulations to preliminarily evaluate the stability of the potential interaction between puerarin and the key target proteins. Importantly, our study demonstrates for the first time that puerarin can modulate the cGAS-STING signaling pathway in ICH mice, thereby alleviating WMI and BBB disruption.

## 2. Materials and Methods

### 2.1. Assessment of Puerarin’s Therapeutic Efficacy in Mice

#### 2.1.1. ICH Model and Drug Administration

Adult male C57BL/6J mice aged 8 weeks and weighing 25–28 g were selected for the experiment and were purchased from Shanghai Jihui Laboratory Animal Care Co., Ltd. (Shanghai, China). A total of 72 mice were used in three independent experiments to evaluate the therapeutic efficacy of Puerarin. All experimental protocols had been approved by the Institutional Animal Care and Use Committee at Tongji University (Ethical number: No. 2024-DW-(018)). Mice were housed in a room-temperature, pathogen-free environment with a 12 h light–dark cycle and had free access to food and water. The ICH mouse model was established in accordance with previously described methodologies [16]. In summary, male mice were anesthetized using 4% isoflurane delivered in 100% oxygen until complete loss of consciousness was achieved. Following anesthesia, Collagenase IV was injected into the striatum (AP 0.2, ML 2.3, DV 3.5) at a rate of 0.1 μL/min using a stereotaxic instrument. In the sham group, an identical procedure was performed, with the exception that a saline solution was administered.

Puerarin (PU) (Cat. No. P5555, Sigma, St. Louis, MO, USA) was prepared at 50 mg/mL in DMSO (Cat. No. D5879, Sigma, St. Louis, MO, USA) [11]. Mice were randomly assigned to the sham or ICH groups and then treated as follows: (1) Sham group: mice subjected to sham surgery received intraperitoneal injections of vehicle (2.5% DMSO in 0.9% NaCl) as the control; (2) Sham + Puerarin group (Sham + Pu): sham-operated mice were administered puerarin (200 mg/kg/day) intraperitoneally for 14 consecutive days after ICH; (3) ICH group: mice that underwent ICH surgery received intraperitoneal injections of vehicle (2.5% DMSO in 0.9% NaCl); (4) ICH + Low-dose puerarin group (ICH + L-Pu): ICH mice were treated intraperitoneally with 50 mg/kg puerarin daily for 14 days after ICH; (5) ICH + High-dose Puerarin group (ICH + H-Pu): ICH mice received intraperitoneal injections of 200 mg/kg puerarin for 14 consecutive days post-ICH to evaluate the therapeutic effect of puerarin on ICH-induced injury.

#### 2.1.2. Neurological Deficit Scoring and Behavioral Test

Neurobehavioral evaluations were performed by investigators blinded to group allocation at days 0, 1, 3, 5, 7, and 14 post-ICH using three tests. The mNSS served as a comprehensive evaluation tool. This score has a scoring range from 0 to 18, where a score of 0 reflects normal neurological function, and higher scores represent increasing severity of impairment. Injury severity was classified as mild (scores 1–6), moderate (scores 7–12), and severe (scores 13–18).

The forelimb placing test measured contralateral paw placement in response to vibrissae stimulation. Performance was recorded over 5 consecutive trials. In the corner turn test, the proportion of right turns made to exit a 30° corner was recorded in 10 trials. An asymmetry score was calculated using the formula [(R − L)/(R + L + B)] × 100%, where R is right turns, L is left turns, and B is bilateral turns. This score quantitatively evaluates motor asymmetry and lateralized neurological impairment, serving as critical indicators of stroke-induced deficits and therapeutic efficacy. Forelimb asymmetry was also assessed in the cylinder test. In the Rotarod test, mice were placed in a rotating drum (Rota-Rod R/S, Model RS-01, RWD Life Science, Shenzhen, China) that accelerated from 5 to 40 rpm within 5 min. Each mouse underwent three consecutive behavioral tests daily for 3 days before surgery, and on the third day after surgery. Additionally, there was a 15 min interval between each test. On day 14 post-ICH, the open field test (OFT) was performed; during this test, spontaneous activity was recorded for 5 min using Smart3.0 software. Both the travel time in the center and the total distance traveled were measured and analyzed.

#### 2.1.3. Evaluation of Cerebral Blood Flow (CBF)

LSCI: The RWD Laser Speckle Imaging System (RFLSI III, RWD) was used to monitor CBF according to methods detailed in previously study [17]. Mice were anesthetized with 4% isoflurane. Following anesthesia, the skull was surgically exposed and cleaned with 3% hydrogen peroxide (H_2_O_2_). Next, the laser was positioned 10 cm above the skull to prepare for CBF measurements. One hour after ICH, CBF measurements were obtained, and local CBF was calculated and analyzed using RWD LSCI software. (RWD Life Sciences, V01.00.05.18305).

#### 2.1.4. Assessment of BBB Permeability

Two weeks after model establishment and corresponding treatments, mice were randomly selected from each group. The animals were anesthetized with 4% isoflurane inhalation, and 2% Evans blue (EB) solution (4 mL/kg) was injected into the tail vein 2 h before euthanasia. After 2 h of circulation, the chest was rapidly opened to expose the heart. A perfusion needle was inserted into the aortic root via the left ventricle, and the right atrial appendage was incised as an outlet. Saline was rapidly perfused until the effluent from the right atrium became clear, after which the skull was quickly opened to harvest the brain. To quantify Evans blue leakage, mouse brains were longitudinally cut into two hemispheres along the midline and the weight of each hemisphere was determined. The right hemisphere was then immersed in formamide solution at a ratio of 10 mL/g, homogenized, and incubated in a 60 °C water bath for 24 h. Subsequently, the homogenates were centrifuged at 10,000 rpm for 20 min in a 4 °C high-speed centrifuge. In total, 200 μL of supernatant from each tube was transferred to a 96-well plate, and optical density (OD) was measured at 620 nm using a microplate reader. Finally, the EB content in brain tissues was calculated based on the EB standard curve. The formula for Evans Blue extravasation is as follows: EB extravasation (OD620/g tissue) = (OD620 (sample) − OD620 (blank))/Tissue wet weight (g). EB content (μg/g tissue) = [EB] × V/W, where [EB] is the EB concentration (μg/mL) obtained from the standard curve, V is the total volume of formamide used for extraction (mL), and W is the wet weight of the tissue (g). This protocol follows the validated method of Wei et al. [18].

#### 2.1.5. Assessment of WMI

MRI: As previously described [19], mice were anesthetized with 4% isoflurane at a respiratory rate of 50–100 breaths per minute. Magnetic resonance imaging (MRI) was then performed using a horizontal bore 9.4T/30 cm MR (uMR 9.4T, United Imaging Life Science Instrument, Wuhan, China). A combination of an 86 mm inner diameter (ID) volume coil for radio frequency (RF) transmission and a custom- designed dedicated RF coil for RF reception was used. Imaging parameters were as follows: echo time, 19 ms; repetition time, 1500 ms; field of view, 15 × 15 mm; and slice thickness, 0.5 mm. Fractional anisotropy (FA) values were quantitatively analyzed using ExploreDTI (version 4.8.6) running on MATLAB R2020a (version 9.8, MathWorks, Natick, MA, USA).All quantitative analyses were performed by an investigator who was blinded to the group allocation.

TEM: Ultrastructural analysis of myelinated axons in the corpus callosum was performed using transmission electron microscopy (TEM). Mice were transcardially perfused with 0.1 M PBS, followed by a fixative solution containing 2.5% glutaraldehyde and 2% paraformaldehyde in 0.1 M PBS. The corpus callosum was carefully dissected and post-fixed in the same fixative at 4 °C for 24 h. After fixation, tissue samples were washed with PBS and post-fixed in 1% osmium tetroxide for 2 h. Samples were then dehydrated through a graded ethanol series and embedded in epoxy resin. Ultrathin sections (~70 nm) were prepared using an ultramicrotome, mounted on copper grids, and stained with uranyl acetate and lead citrate for electron microscopic analysis.

The stained sections were examined using a transmission electron microscope (HT7800, Hitachi, Tokyo, Japan) operated at 80 kV. For each mouse, at least 100 clearly defined, circular myelinated axons from the corpus callosum were randomly selected and imaged at 5000× magnification. Axon and fiber diameters were quantitatively measured using ImageJ software (version 1.53c, NIH, Bethesda, MD, USA). The g-ratio for each axon was calculated as the ratio of axon diameter to fiber diameter. The average g-ratio and myelin thickness per animal were subsequently used for statistical analysis.

### 2.2. Transcriptome Analysis and Machine Learning to Identify Target Gene

#### 2.2.1. Study Design and Data Collection

We obtained Gene expression data from the GEO database (GSE228222). Dataset included 6 samples (3 sham controls and 3 ICH mouse models), profiling the expression of 18,173 genes. Gene expression levels, derived from RNA sequencing, were quantified using the FPKM metric.

#### 2.2.2. Transcriptome Sequencing and Differential Expression Gene (DEG) Analysis

ICH datasets (GSE228222) were analyzed using the GEO query package in R software (version 4.5.0), followed by preprocessing steps: background correction, normalization, and log2 transformation. For genes with multiple probes, expression values were averaged to yield a single representative measure per gene. DEGs were identified using the “limma” package, applying thresholds of |log2FoldChange| > 1 and an adjusted *p*-value < 0.05.

#### 2.2.3. WGCNA Was Employed to Identify Gene Modules Associated with ICH

To explore gene interactions, we used WGCNA package [20] to build a weighted gene co-expression network. The top 25% most variable genes were selected, and outlier samples were excluded to enhance network reliability. The adjacency matrix was subsequently converted into a topological overlap matrix (TOM), and the corresponding dissimilarity (1-TOM) was calculated. Gene modules were delineated through hierarchical clustering based on TOM dissimilarity, with a minimum module size of 60 genes. Module eigengenes (MEs) were calculated, and modules significantly linked to clinical traits were identified. In key modules, we assessed module membership and gene significance to prioritize relevant genes. We visualized networks within significant modules to provide further insights.

#### 2.2.4. Integration of Puerarin Targets and ICH-Related Genes

We use PubChem database (https://pubchem.ncbi.nlm.nih.gov/, accessed on 23 March 2025) to obtain the chemical structure of puerarin. Possible therapeutic genes of puerarin were estimated employing computational platforms, including the Similarity Ensemble Approach (SEA; https://sea.bkslab.org, accessed on 25 March 2025), PharmMapper (https://www.lilab-ecust.cn/pharmmapper/, accessed on 25 March 2025), the Traditional Chinese Medicine Systems Pharmacology Database and Analysis Platform (TCMSP; https://tcmsp-e.com, accessed on 25 March 2025), and SwissTargetPrediction (https://www.swisstargetprediction.ch, accessed on 25 March 2025). To enhance reliability, outputs from these databases were combined, with redundant entries eliminated. To determine prospective targets underlying drug effectiveness, the DEG, WGCNA, and predicted drug targets of puerarin were integrated. The “Compound-Disease Target” Venn diagram was generated by intersecting ICH-related genes with predicted puerarin targets (obtained from https://www.bioinformatics.com.cn, accessed on 25 March 2025), and the intersections were visualized using a Venn diagram.

#### 2.2.5. Screening of Hub Targets Using Machine Learning Algorithms

To identify discriminative molecular biomarkers for ICH, we implemented a dual-model machine learning strategy combining Ridge Regression and Logistic Regression, both employing L2 regularization to enhance model robustness and generalizability. All analyses were conducted in R software using the glmnet package (version 4.5.0). For Ridge Regression, the model was fitted with alpha = 0, and the lambda hyperparameter was optimized via leave-one-out cross-validation (LOOCV). Feature importance was assessed based on the absolute values of the regression coefficients. Similarly, the Logistic Regression model (family = “binomial”, alpha = 0) was tuned using LOOCV. The use of Ridge regularization in both models ensured stable and interpretable feature ranking.

#### 2.2.6. Biological Function Analysis

Gene Ontology (GO) and Kyoto Encyclopedia of Genes and Genomes (KEGG) enrichment analyses were performed using the clusterprofiler package in R to identify the biological roles and pathways associated with the candidate genes. GO annotations were further examined, including biological processes (series of molecular events), cellular components (subcellular locations), and molecular functions (gene-associated biochemical activities). Enrichment was considered significant when the adjusted q-value was less than 0.05.

### 2.3. Network and Interaction Validation

#### 2.3.1. PPI Network Construction

We constructed the protein–protein interaction (PPI) network of candidate targets utilizing the STRING database (version 12.0; https://string-db.org/, accessed on 16 June 2025) and established a confidence score exceeding 0.4 as the threshold. The resulting network was subsequently transferred into Cytoscape software (version 3.9.1) for graphical representation and examination. Using the Network Analyzer tool, we calculated node degree centrality to facilitate the determination of hub genes. Subsequently, we employed the CytoHubba plugin to extract the top 20 highest-ranked genes and establish a core subnetwork of key targets.

#### 2.3.2. Molecular Docking

Ligand structure was first acquired from the PubChem database (http://pubchem.ncbi.nlm.nih.gov/, accessed on 16 June 2025) and energetically optimized using ChemOffice. Next, the protein crystal structure was downloaded from the RCSB PDB (Protein Data Bank, http://www.rcsb.org/, accessed on 16 June 2025) and prepared by removing water molecules and phosphate ions in PyMOL (version 3.0.5). Subsequently, molecular docking simulations were performed with AutoDock Vina 1.5.6 to investigate the interactions among the proteins and ligands, utilizing a defined grid box. Finally, the optimal pose was selected based on binding affinity and visualized using Discovery Studio 2019.

#### 2.3.3. Molecular Dynamics Simulation

We conducted molecular dynamics simulations utilizing GROMACS 2022. Protein force field parameters were produced using the pdb2gmx utility of GROMACS according to the CHARMM36 force field, while ligand parameters were obtained from the AutoFF web server using the GAFF2 force field with partial charges assigned by the RESP method. The system was submerged in a TIP3P water box and equilibrated with 0.15 M NaCl. Energy minimization was accomplished employing steepest descent and conjugate gradient techniques. Production simulations were executed for 100 ns in the NPT ensemble at 310 K and 1 bar. Structural stability and binding interactions were assessed by calculating the root mean square deviation (RMSD), root mean square fluctuation (RMSF), hydrogen bonds, radius of gyration (Rg), Solvent Accessible Surface Area (SASA), and Molecular Mechanics energies combined with the Poisson-Boltzmann Surface Area(MM-PBSA) binding free energy using standard GROMACS tools.

### 2.4. Validation of Puerarin Targets and Signaling Pathways

#### 2.4.1. Western Blot

White matter tissues from mice were lysed in RIPA buffer (89900, Thermo Fisher, Waltham, MA, USA). Protein concentrations were quantified employing a BCA protein assay kit (Solarbio Biotechnology, Beijing, China). Equivalent quantities of protein were resolved using SDS-PAGE gel (Epizyme, Shanghai, China) and transferred onto PVDF membranes (Millipore, Billerica, MA, USA). Following this, membranes were blocked with 5% non-fat milk in TBST for 1 h at room temperature. The blocked membranes were then incubated overnight at 4 °C with primary antibodies: rabbit anti-MBP (1:2000, 10458-1-AP, Proteintech, Wuhan, China), rabbit anti-STING (1:1000, 19851-1-AP, Proteintech, Wuhan, China), rabbit anti-cGAS (1:2000, PQA3430, Abmart, Berkeley Heights, NJ, USA), rabbit anti-p-TBK1 (1:1000, 5483, Cell Signaling Technology, Danvers, MA, USA), rabbit anti-TBK1 (1:1000, 3504, Cell Signaling Technology), mouse anti-Beta Actin (1:5000, 66009-1-Ig, Proteintech, Wuhan, China) and rabbit anti-GAPDH (1:5000, 5174, Abcam, Cambridge, UK). After incubation, membranes were washed three times with TBST. Next, they were incubated with HRP-conjugated secondary antibodies (1:5000) for 1 h at room temperature. Protein bands were visualized using a chemiluminescence detection kit (Thermo Fisher Scientific, Waltham, MA, USA) and imaged on a ChemiDoc XRS+ system (Tanon 5200CE, Shanghai, China). Finally, all Western blot data were normalized to GAPDH, and band intensities were measured employing ImageJ.

#### 2.4.2. Immunofluorescence

Mouse brains were extracted from the skull and subsequently fixed in 4% PFA at 4 °C for 1–2 days, followed by sequential dehydration with 20% and 30% sucrose solution (dissolved in 0.1 M PBS) for 24 h, respectively. The dehydrated brains were embedded in OCT (Sakura Finetek, Torrance, CA, USA) and sectioned at a thickness of 30 μm using a cryostat (FS800A, RWD Life Science Co., Ltd, Shenzhen, China).

For immunostaining, frozen brain sections were chosen. These were preincubated in 0.2% Triton X-100 for 1 h at RT, then blocked with 0.05% Triton X-100 and 5% Goat serum in PBS for 1 h at RT. Sections were rinsed in PBS. Neurons or sections were moved into primary antibody mixture (Rabbit anti-MBP (1:300,10458-1-AP, Proteintech); Rabbit anti-Olig2 (1:200, ab254026, Abcam); Mouse anti-CC1 monoclonal antibody [1:300, OP80, Millipore] in PBS with 0.2% Triton-X100 and incubated overnight at 4 °C. Tissues were rinsed in PBS three times and incubated with secondary antibody solution (Goat anti-Rabbit 488, Goat anti-Rabbit 568, Goat anti-Mouse 568, 1:500, Invitrogen, Carlsbad, CA, USA) in PBS for 2 h at RT. Nuclei were stained with DAPI (62247, 1:1000, Thermo Fisher Scientific). Apoptotic cells in the neurons or sections were detected using a TUNEL assay kit (E-CK-A320, Elabscience, Wuhan, China) in accordance with the manufacturer’s guidelines. Sections were visualized with a Leica MICA slide scanning microscope. Confocal images were captured with an Olympus FV31S-SW confocal laser scanning microscope (Olympus, Tokyo, Japan). Images were evaluated in ImageJ.

### 2.5. Statistical Analysis

All statistical analyses were performed using GraphPad Prism 9.5.0 and SPSS 26.0. Data are presented as mean ± standard deviation. Normality was assessed using the Shapiro–Wilk test. For comparisons between two groups, Student’s *t*-test was applied to normally distributed data, whereas the Mann–Whitney U test was used for non-normally distributed data. Comparisons among multiple groups were performed using one-way ANOVA followed by Tukey’s post hoc test. A two-tailed *p*-value < 0.05 was considered statistically significant.

## 3. Results

### 3.1. Puerarin Alleviated Neurological Deficits in ICH Mice

**Figure 1:**
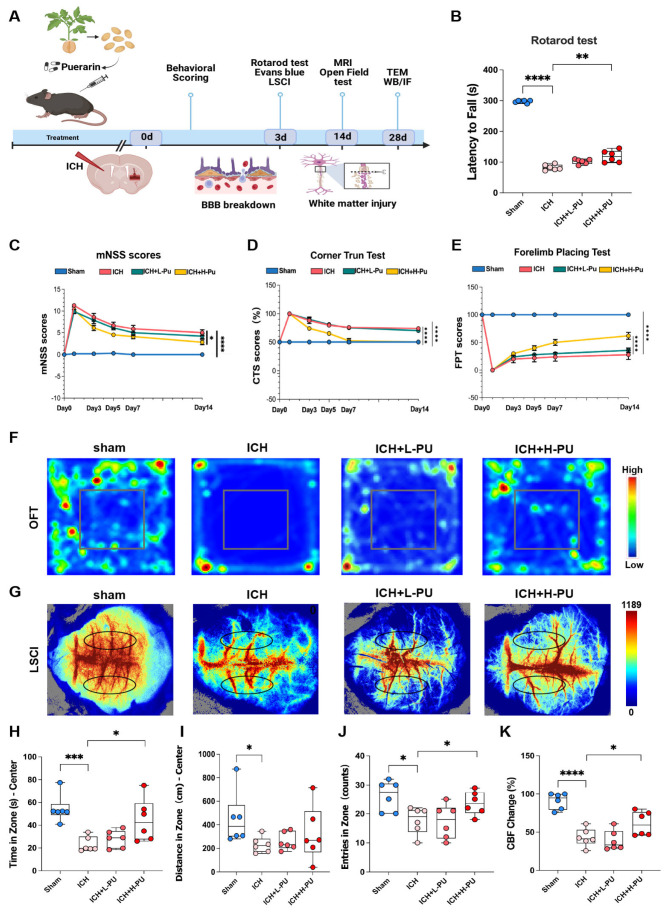
Puerarin ameliorates neurological and behavioral deficits following ICH. (**A**) Schematic illustration of the experimental timeline for behavioral assessments in the ICH mouse model. Puerarin or vehicle (DMSO) was administered intraperitoneally (i.p.) once daily for 14 consecutive days. (**B**) Latency to fall in the rotarod test was assessed on day 3 after ICH (*n* = 6 per group). (**C**) Neurological deficits were evaluated using the modified Neurological Severity Score (mNSS) on day 0, 1, 3, 5, 7, and 14 after ICH (*n* = 6 per group). (**D**) Corner turn test (CTS) scores (%) among the four groups at the indicated time points (*n* = 6 per group). (**E**) Forelimb placing test (FPT) scores (%) among the four groups at the indicated time points (*n* = 6 per group). (**F**,**H**–**J**) Open-field test results. Representative heat maps and quantitative analyses show time spent in the center zone, number of center entries, and total travel distance among the four groups after ICH (*n* = 6 per group). (**G**,**K**) Representative cerebral blood flow (CBF) images acquired on day 3 after ICH. The black circle indicates the region of interest (ROI) (*n* = 6 per group). Data are presented as mean ± SEM. Statistical significance was determined by one-way ANOVA followed by Tukey’s post hoc test. * *p* < 0.05, ** *p* < 0.01, *** *p* < 0.001, **** *p* < 0.0001.

To investigate the role of puerarin in ICH progression, we established a collagenase IV–induced mouse model and performed a series of neurological and behavioral assessments (Figure 1A). In the rotarod test, we observed a significantly reduced latency to fall in ICH mice compared with the sham group (*p* < 0.0001). This impairment was markedly ameliorated by high-dose puerarin (200 mg/kg/day, ICH + H-Pu, (*p* < 0.01), but not by low-dose puerarin (50 mg/kg/day, ICH + L-Pu) (Figure 1B). We found that the modified Neurological Severity Score (mNSS) was significantly elevated at 24 h post-ICH and was markedly reduced by both low-dose and high-dose puerarin treatment, with no significant difference between the two dosage groups (Figure 1C). By day 14, we observed significantly impaired turning symmetry in the corner test in ICH mice compared with the sham group (*p* < 0.0001). Notably, this deficit was markedly improved in the ICH + H-Pu group compared with the ICH group (*p* < 0.0001) (Figure 1D). Consistently, the forelimb placement test revealed severe left forelimb dysfunction in the ICH group relative to the sham group, which was ameliorated by high-dose puerarin treatment, as shown by improved performance in the ICH + H-Pu group (Figure 1E). In the open field test, ICH mice exhibited reduced center zone time (*p* < 0.001) and entries compared with sham controls (*p* < 0.05). While ICH + H-Pu mice spent more time in the center zone and made more center entries than ICH mice (*p* < 0.05), no significant improvement was observed in the ICH + L-Pu group. The total travel distance in the center zone did not differ significantly among all groups (*p* = 0.3618; Figure 1F,H–J). Laser speckle contrast imaging (LSCI) further confirmed the ICH-induced reduction in cerebral blood flow (CBF) relative to the sham group (*p* < 0.0001), and revealed that CBF was significantly improved in the ICH + H-Pu group compared with the ICH group (*p* < 0.05), an effect not observed with low-dose puerarin treatment (Figure 1G,K). Collectively, these results indicate that ICH induces robust neurological dysfunction and that high-dose puerarin effectively alleviates these deficits in mice.

### 3.2. Puerarin Attenuated WMI and BBB Disruption in ICH Mice

**Figure 2:**
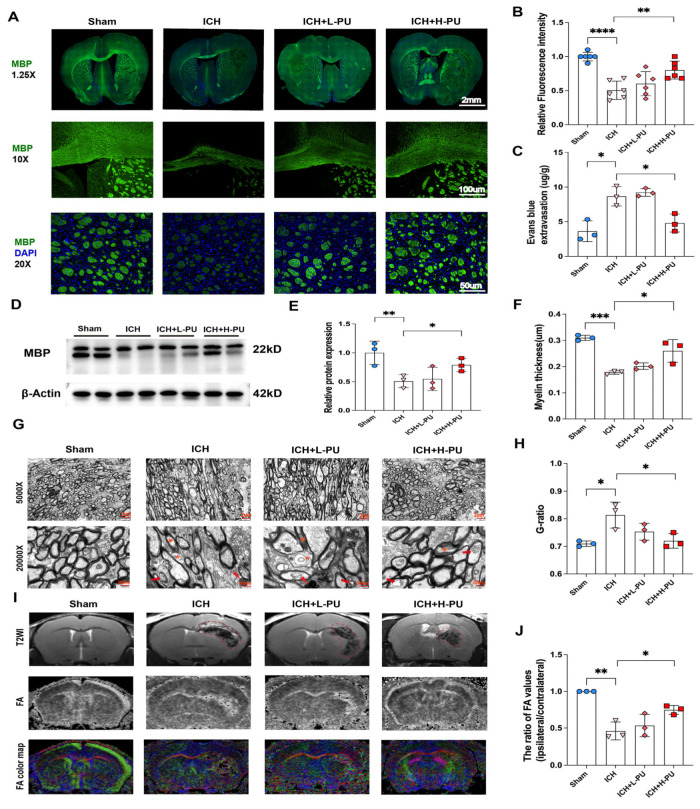
Puerarin attenuates white matter injury and blood–brain barrier disruption after ICH. (**A**) Representative immunofluorescence images of myelin basic protein (MBP) staining in the corpus callosum and striatum from each group. Scale bars: 2 mm (upper panel), 100 μm (middle panel), and 50 μm (lower panel). (**B**) Quantitative analysis of MBP immunofluorescence intensity in the corpus callosum and striatum. (**C**) Effect of puerarin treatment on Evans blue extravasation at 72 h after ICH. Evans blue content was quantified by measuring absorbance at 620 nm and normalized to tissue weight (ug/g). (**D**) Western blot analysis of MBP expression in the white matter from each group. The original western blot images can be found in Figure S3. (**E**) Quantitative analysis of MBP levels normalized to β-actin. (**F**) Quantitative analysis of myelin sheath thickness in the corpus callosum. (**G**) Representative transmission electron micrographs of the corpus callosum from ICH model mice. Scale bars: 2 μm (upper panel) and 500 nm (lower panel). Demyelinated axons are indicated by red asterisks (*). Newly formed axons surrounded by loose myelin are indicated by red stars. Swollen mitochondria and excessive myelin debris are indicated by red arrows. (**H**) Quantitative analysis of the g-ratio in the corpus callosum. (**I**) Representative T2-weighted images (upper panel), diffusion tensor images (middle panel), and color-coded fractional anisotropy (FA) maps (lower panel) from the collagenase-induced ICH model. The red circle indicates the hematoma area. (**J**) Quantitative analysis of FA value ratios. Data are presented as mean ± SEM. Statistical significance was determined by one-way ANOVA followed by Tukey’s post hoc test (* *p* < 0.05, ** *p* < 0.01, *** *p* < 0.001, **** *p* < 0.0001). In all bar graphs, the overlaid geometric shapes represent individual biological replicates from different experimental groups: circles for the Sham group, triangles for the ICH group, diamonds for the ICH+ Low-dose Puerarin group(ICH+L-PU), and squares for the ICH + High-dose Puerarin (ICH + H-Pu) group.

Prompted by our finding that puerarin (Pu) attenuated neurological dysfunction following ICH, we next investigated its effects on white matter injury (WMI) and blood–brain barrier (BBB) disruption. We first assessed BBB integrity. Evans blue extravasation (Figure 2C) and the corresponding OD620 values (Supplementary Figure S2) in the ipsilateral hemisphere were significantly higher in ICH mice than in sham-operated mice, confirming severe BBB disruption. In contrast, high-dose puerarin treatment (H-Pu) significantly reduced this extravasation compared to the ICH group, suggesting it effectively attenuated BBB permeability (*p* < 0.05). We then evaluated WMI. Immunofluorescence analysis of the corpus callosum (CC) at day 28 post-ICH revealed a significant decrease in the mean fluorescence intensity of myelin basic protein (MBP) in the ICH group compared to the sham group, which was attenuated by high-dose puerarin (ICH + H-Pu group; *p* < 0.01 vs. ICH group) (Figure 2A,B). Consistent with this, Western blot analysis confirmed that MBP expression, a major structural component of myelin, was significantly reduced in the ICH group relative to sham (*p* < 0.01) and was significantly increased in the ICH + H-Pu group compared with the ICH group (*p* < 0.05; Figure 2D,E). We further assessed ultrastructural changes using transmission electron microscopy (TEM). TEM analysis of the CC at day 28 post-ICH revealed that the ICH group exhibited a marked reduction in myelin thickness (*p* < 0.001) and a significant increase in the g-ratio (*p* < 0.05) compared with the sham group (Figure 2F–H). In contrast, these ultrastructural alterations were substantially reversed in the ICH + H-Pu group, which showed increased myelin thickness (*p* < 0.05) and a reduced g-ratio (*p* < 0.05) compared with the ICH group. The ICH + L-Pu group also showed a trend toward a reduced g-ratio (Figure 2H). Finally, magnetic resonance imaging (MRI) on day 14 post-ICH visualized these changes. Representative T2-weighted images, diffusion tensor imaging (DTI), and color-coded fractional anisotropy (FA) maps are shown in Figure 2I. Hematoma formation was evident in the right hemisphere of ICH mice, whereas no lesions were detected in the sham group, highlighting the structural and microstructural differences across groups. All images were co-registered to a standardized template, and we carefully defined regions of interest (ROIs). FA maps in the ICH group illustrated the three-dimensional orientation of diffusion tensors. Compared to the homogeneous and ordered fiber architecture observed in the sham group, while the bilateral corpus callosum (CC) remained largely intact, the right internal capsule (IC) exhibited pronounced damage in ICH mice. We applied standardized bilateral CC and IC ROIs to the template, taking voxel size and anatomical location into account. FA values, normalized on a scale from 0 to 1, were quantified for each subgroup. Consistent with the observed structural damage, FA ratios were markedly reduced in the ipsilateral regions of the ICH group compared with the sham group, with the IC showing a more pronounced decrease (Figure 2J). The ICH + L-Pu and ICH + H-Pu groups also showed reduced FA ratios relative to sham. Notably, the ICH + H-Pu group exhibited a significantly higher FA ratio than the ICH group (*p* < 0.05), indicating improved preservation of white matter fibers and attenuated WMI. In contrast, we did not observe a significant difference between the ICH + L-Pu and ICH groups.

### 3.3. Therapeutic Targets of Puerarin for Alleviating White Matter Injury After ICH

**Figure 3:**
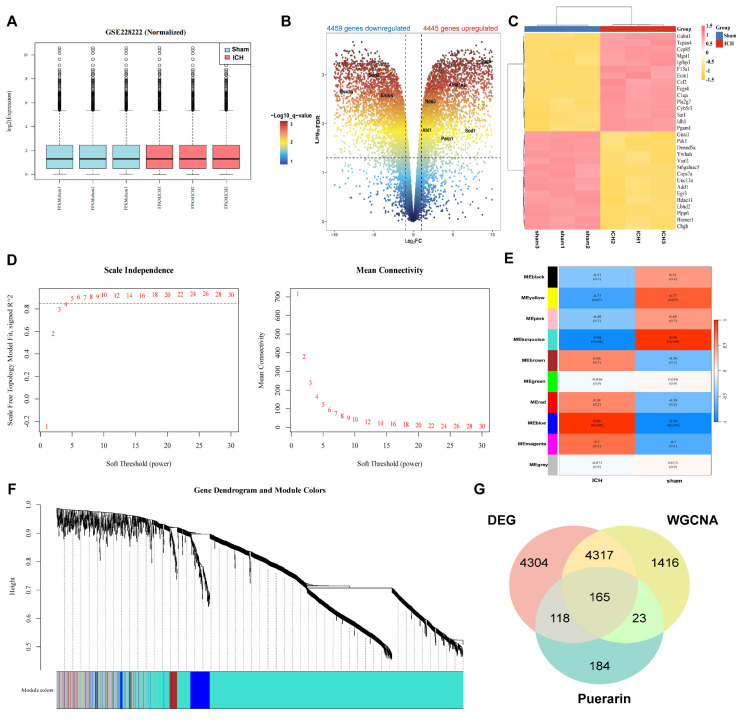
Therapeutic targets of puerarin for alleviating white matter injury after ICH (**A**) Box plot of gene expression after batch correction. (**B**) Volcano plot showing differentially expressed genes identified from the GSE228222 dataset. (**C**) Heatmap illustrating the expression profiles of intersecting candidate genes. (**D**) Analysis of scale-free topology fit index and mean connectivity across a range of soft-thresholding powers to determine the optimal power for constructing a scale-free gene co-expression network. (**E**) Module–trait relationships are displayed as a color-coded heatmap, with correlation coefficients indicating associations between module eigengenes and ICH traits. (**F**) Gene clustering dendrogram with module assignment, showing the distribution of genes into distinct co-expression modules. (**G**) Venn diagram illustrating the overlap among puerarin targets, ICH-related genes, and genes identified from DEG and WGCNA analyses. Puerarin targets are shown in green, while DEG- and WGCNA-derived genes are shown in red and yellow, respectively.

We first identified the potential targets of puerarin by integrating data from five databases (TCMSP, SEA, PharmMapper, SwissTargetPrediction, and TargetNet). After removing duplicate entries, a total of 490 unique puerarin-related targets were obtained for subsequent analyses. Next, we analyzed the GSE228222 dataset to identify genes associated with ICH. After correction for batch effects (Figure 3A), we identified 8904 differentially expressed genes (DEGs) between the sham and ICH groups, including 4445 upregulated and 4459 downregulated genes. The distribution of DEGs was visualized using a volcano plot (Figure 3B), and their expression patterns were further illustrated by a clustered heatmap (Figure 3C). We then performed weighted gene co-expression network analysis (WGCNA) on 18,173 genes. Using a soft-thresholding power of β = 4, a scale-free co-expression network was constructed (Figure 3D), and genes were grouped into 10 distinct modules. Module–trait correlation analysis identified the turquoise module as the most strongly associated with ICH (Figure 3E,F), yielding 5921 module-specific genes. Finally, intersection analysis of puerarin targets, GEO-derived DEGs, and genes from the turquoise WGCNA module resulted in 165 overlapping genes, which were defined as candidate therapeutic targets of puerarin in ICH (Figure 3G).

### 3.4. Machine Learning Screening of Core Targets and Enrichment Analysis

**Figure 4:**
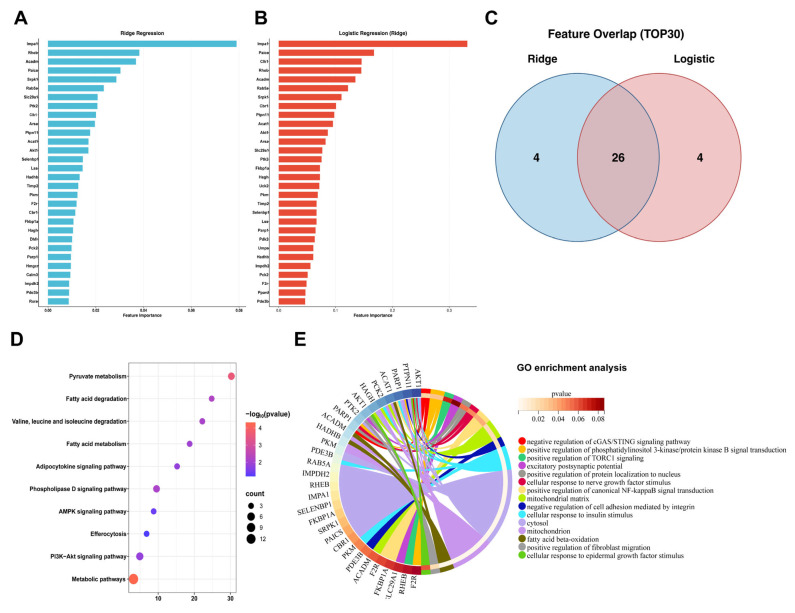
Machine learning predicted hub genes in ICH. (**A**) Feature importance of genes derived from Ridge regression. This bar plot displays the feature importance of each gene feature as determined by the Ridge regression algorithm. The features are ranked along the horizontal axis, illustrating how L2 regularization shrinks coefficients and identifies a stable set of predictors. (**B**) Feature importance of genes derived from logistic regression. This bar plot shows the feature importance of each gene feature selected by the logistic regression model in predicting the binary outcome. Features are similarly ranked by their importance scores along the horizontal axis. (**C**) Intersection of selected features from both algorithms. The Venn diagram (or similar intersection plot) illustrates the overlap between the feature sets identified by Ridge regression (blue) and logistic regression (red). (**D**) KEGG pathway enrichment analysis of the overlapping genes. The bubble plot visualizes the significantly enriched KEGG pathways. The color gradient of the bubbles represents the statistical significance (−log_10_(*p* value) or adjusted *p* value), while the bubble size corresponds to the number of enriched genes mapped to each pathway. (**E**) GO enrichment analysis of the overlapping genes. The circular chord diagram depicts the relationships between the selected genes (arranged on the circle’s periphery) and their significantly enriched Gene Ontology (GO) terms (biological process, molecular function, or cellular component, displayed in arcs or sectors). The chords connecting the features to the terms illustrate the annotation mapping.

We applied a dual-model machine learning framework combining Ridge regression and logistic regression to screen core targets associated with intracerebral hemorrhage (ICH) (Figure 4A,B). Using a TOP30 feature selection strategy, we intersected the top 30 ranked features from each model and identified 26 shared hub genes (Figure 4C). Among these hub genes, *AKT1* and *PARP1* were consistently ranked highly by both models. We then performed functional enrichment analysis of the 26 hub genes. Kyoto Encyclopedia of Genes and Genomes (KEGG) and Gene Ontology (GO) analyses revealed that these genes were mainly enriched in biological processes and pathways related to the negative regulation of cGAS–STING signaling, positive regulation of PI3K/PKB and TORC1 signaling, fatty acid oxidation, cellular response to nerve growth factor stimulus, and mitochondrial function (Figure 4D,E). These enrichment results indicated that the identified hub genes were closely associated with immune regulation, energy metabolism, and neural survival following ICH.

### 3.5. Integrative Analysis of Therapeutic Targets and Pharmacologic Networks in ICH

**Figure 5:**
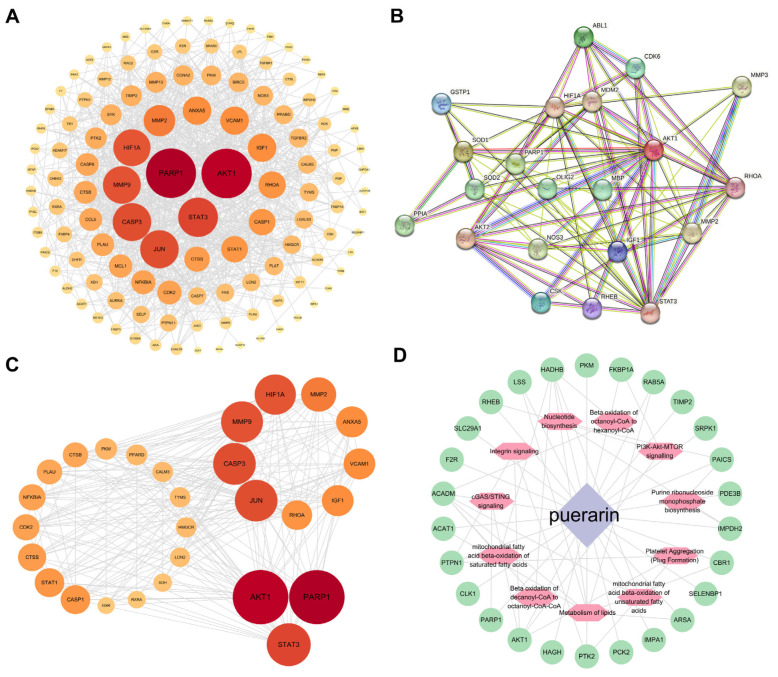
Integrative analysis of therapeutic targets and pharmacologic networks in ICH. (**A**) PPI network of hub targets shared between puerarin and WMI post-ICH. This network diagram visualizes high-confidence interactions among hub genes identified from the common targets of puerarin and WMI. Genes are represented as nodes, in which larger node sizes and darker colors indicate higher degree values, highlighting genes that are topologically central and potentially functionally crucial within the network. Interactions were derived from the STRING database using a high-confidence score threshold. (**B**) Specific interaction network between puerarin targets and key myelination-related proteins (OLIG2 and MBP). This subnetwork, generated using the STRING database, focuses on direct and indirect interactions between the predicted drug targets of puerarin and two critical proteins involved in oligodendrocyte function and myelination, namely OLIG2 and MBP. It was aimed at elucidating potential direct regulatory points of puerarin in the repair of damaged white matter. (**C**) Detailed view of the top hub targets within the shared PPI network. This panel provides a focused representation of proteins with the highest connectivity from the global network shown in (**A**). (**D**) Functional modules of the PPI network related to biological processes involved in WMI repair. This network visualization, constructed and analyzed using Cytoscape, represents the results of GO enrichment analysis and maps the common targets onto a PPI network in which nodes are color-coded or clustered according to their significant enrichment in specific GO biological process terms.

We constructed a protein–protein interaction (PPI) network using Cytoscape, and targets were further filtered based on the median value. As shown in Figure 5A,C, nodes with larger circle sizes and redder colors represented higher centrality values. In addition, a PPI network illustrating interactions between puerarin-related targets and the white matter–associated proteins Olig2 and MBP was generated using the STRING database (Figure 5B). We next performed GO enrichment analysis to further characterize the biological functions of these targets. The results showed that puerarin-associated genes involved in myelin repair after ICH were significantly enriched in biological processes related to cGAS/STING signaling, fatty acid oxidation and metabolic pathways, mitochondrial fatty acid β-oxidation of saturated fatty acids, and platelet aggregation (Figure 5D). Complementarily, KEGG enrichment analysis identified the top 10 significantly enriched pathways (Figure 4D), among which “fatty acid degradation” and “pyruvate metabolism” were the most prominent. These pathways were closely associated with energy metabolism in myelin and oligodendrocytes, processes that are critical for maintaining white matter integrity. Disruption of these metabolic pathways has been reported to contribute to white matter abnormalities in aging, stroke, and multiple sclerosis [12,13,14].

### 3.6. Molecular Docking Analysis

**Figure 6:**
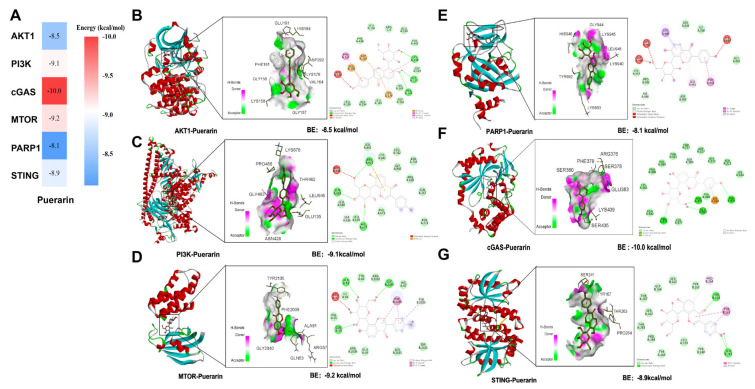
Molecular docking of puerarin with six potential targets in the PI3K/AKT1/mTOR and PARP1/cGAS/STING pathways. (**A**) Heatmap of predicted docking scores summarizing the binding affinities (kcal/mol) of puerarin to six candidate targets. Targets are PI3K, AKT1, and mTOR in the PI3K/AKT1/mTOR pathway, and PARP1, cGAS, and STING in the PARP1/cGAS/STING pathway. Warmer colors (red) indicate stronger predicted binding (more negative docking scores). (**B**–**G**) Representative molecular docking visualizations of puerarin with each target: AKT1 (**B**), PI3K (**C**), mTOR (**D**), PARP1 (**E**), cGAS (**F**), and STING (**G**), highlighting hydrogen bonds, hydrophobic interactions, and key residues involved in binding.

We performed molecular docking analyses to evaluate the potential interactions between puerarin and six core targets from the PARP1/cGAS/STING and PI3K/AKT1/mTOR signaling pathways. Molecular docking analysis suggests potential binding of puerarin to PARP1 and AKT1, which may contribute to its anti-inflammatory effects. Puerarin exhibited strong binding affinity to all examined targets under physiological temperature conditions, with binding free energies ranging from −8.1 to −10.0 kcal/mol (Figure 6A; Table 1). Stable hydrogen bonds were formed between puerarin and residues LYS158, GLY157, and VAL164 in AKT1; ARG376, PHE379, SER435, and GLU383 in cGAS; ALA81 and GLY2040 in mTOR; GLN818, ARG821, and HIS677 in PI3K; as well as THR263 and SER241 in STING. In addition, hydrophobic interactions were observed between puerarin and PRO264 in STING and LEU941 in PARP1. Notably, the LYS439 residue of cGAS formed a π–cation interaction with puerarin, while residues LYS362, ASN482, LEU377, TYR436, LYS414, SER213, and ASP227 contributed van der Waals interactions, and SER380 engaged in a π-donor hydrogen bond with puerarin (Figure 6B–G).

### 3.7. Molecular Dynamics (MD) Simulation

#### 3.7.1. Molecular Dynamics Simulation of cGAS–Puerarin Complex

**Figure 7:**
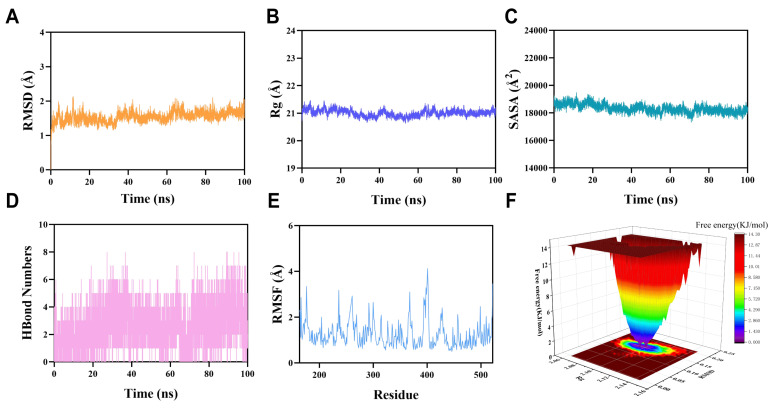
Molecular dynamics simulation of cGAS–puerarin complex. (**A**) Root mean square deviation (RMSD) of the cGAS–puerarin complex during the simulation, indicating overall structural stability relative to the initial docking conformation. (**B**) Radius of gyration (Rg) of the cGAS–puerarin complex, reflecting the global compactness of the protein structure throughout the simulation. (**C**) Solvent-accessible surface area (SASA) of the cGAS–puerarin complex during the simulation, representing changes in solvent exposure upon puerarin binding. (**D**) Time-dependent hydrogen bond (H-bond) analysis of the cGAS–puerarin complex, showing the number of intermolecular hydrogen bonds formed during the simulation. (**E**) Root mean square fluctuation (RMSF) of individual cGAS residues, illustrating residue-level flexibility. (**F**) Free energy landscape (FEL) of the cGAS–puerarin complex, in which the depth and separation of energy basins represent thermodynamic stability and major conformational states.

Molecular docking analysis revealed that the cGAS–puerarin complex exhibited an excellent binding affinity, with a binding energy of −10.0 kcal/mol, which was well below the −7.0 kcal/mol threshold indicative of strong binding. Therefore, 100ns molecular dynamics simulations were carried out for the complexes. As shown in Figure 7A, the RMSD of the cGAS complex reached equilibrium after 70 ns. It eventually fluctuated around 1.8 Å, suggesting high stability of puerarin bound to cGAS. Rg values for cGAS–puerarin complex remained stable, indicating compact protein structures and dynamic stability (Figure 7B). Furthermore, the SASA value for the cGAS protein remained stable throughout the simulation, indicating a compact and stable global structure without substantial unfolding or major conformational rearrangement (Figure 7C). Analysis of intermolecular hydrogen bonds within cGAS–puerarin complex maintained an average of approximately five hydrogen bonds during the simulation, indicating stable binding (Figure 7D). Consistent with these findings, RMSF analysis showed that most residues in the cGAS–puerarin complex displayed low flexibility, with fluctuations mostly below 3 Å, further supporting the high stability of the complex (Figure 7E). Free energy landscape analysis (Figure 7F) revealed that the lowest energy basin was located in a region characterized by low RMSD and low Rg values. Furthermore, MM-PBSA calculations in the last 10 ns of the simulation yielded a total binding free energy of −13.66 kcal/mol for the puerarin–cGAS complex. The main contributing amino acid residues were TYR436, LYS362, PRO306, and VAL360, indicating that puerarin specifically binds to cGAS, thereby exerting neuroprotective effects.

#### 3.7.2. Molecular Dynamics Simulation of AKT1–Puerarin Complex and PARP1–Puerarin Complex

As shown in Supplementary Figure S1A, we observed that the RMSD of the AKT1–puerarin complex reached equilibrium after approximately 90 ns and subsequently fluctuated around 2.3 Å, indicating stable complex formation. Similarly, the PARP1–puerarin complex stabilized after approximately 80 ns, with final RMSD fluctuations of ~1.6 Å (Supplementary Figure S1G), suggesting a stable binding conformation. The profiles of both complexes remained relatively stable throughout the simulation, reflecting maintained structural compactness and dynamic stability. Rg values for AKT1–puerarin complex and the PARP1–puerarin complex remained stable, indicating compact protein structures and dynamic stability (Supplementary Figure S1B,H). Furthermore, the SASA values for AKT1 and PARP1 remained steady throughout the simulation, indicating structural stability and minimal substantial structural alterations (Supplementary Figure S1C,I). Hydrogen bond analysis revealed that the AKT1–puerarin complex maintained between 0 and 6 intermolecular hydrogen bonds during the simulation (Supplementary Figure S1D). In the PARP1–puerarin complex, the number of hydrogen bonds ranged from 0 to 4, with approximately three hydrogen bonds preserved in most simulation frames (Supplementary Figure S1J), supporting stable ligand–protein interactions. Consistent with these findings, RMSF analysis showed that residue-level fluctuations in the AKT1–puerarin complex were generally low, with most values below 3 Å (Supplementary Figure S1E). The PARP1–puerarin complex exhibited even lower RMSF values, with the majority below 2 Å (Supplementary Figure S1K), indicating that binding confers both lower flexibility and greater stability in the binding regions. The conformational sampling was further analyzed using free energy landscape (FEL) projections onto RMSD and Rg (Supplementary Figure S1F,L). MM-PBSA calculations revealed that the total binding free energy of the AKT1–puerarin complex was −9.52 kcal/mol, with key contributing residues including VAL164, PHE161, TYR229, and GLY157. In contrast, the PARP1–puerarin complex exhibited a stronger binding affinity, with a total binding free energy of −15.44 kcal/mol, primarily contributed by residues LEU941, TYR992, LYS940, and HIS946.

### 3.8. Puerarin Alleviated WMI After ICH Was Associated with Reduced Activation of the cGAS–STING Pathway and Improved Myelin Preservation

**Figure 8:**
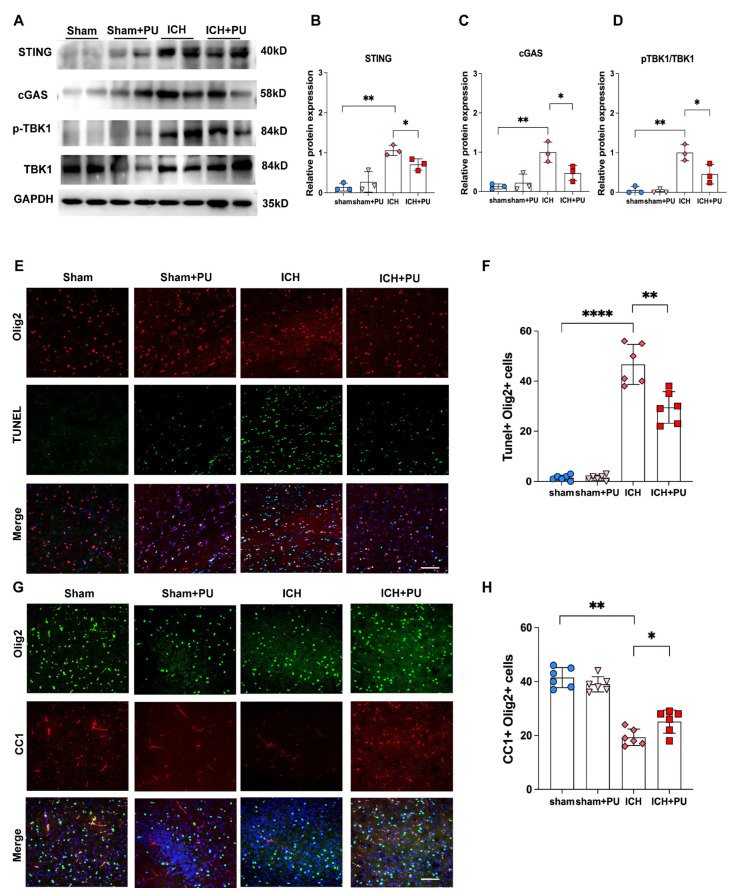
Puerarin alleviated WMI after ICH via blockade of the cGAS-STING pathway. (**A**) Western blot for p-TBK1, TBK1, cGAS and STING. The original western blot images can be found in Figure S4. (**B**–**D**) Quantification of relative STING,cGAS protein levels and the ratio of p-TBK1/TBK1. (**E**) Representative TUNEL staining after puerarin treatment in ICH mice. Representative fluorescent images showing co-localization analysis. Green fluorescence indicates TUNEL staining, and red fluorescence indicates Olig2. (**F**) Quantitative analysis of the proportion of apoptotic oligodendroglia cell. Representative fluorescent images showing co-localization analysis. Green fluorescence indicates Olig2, and red fluorescence indicates CC1. (**G**) Representative immunofluorescent images of Olig2 (green) and CC1 (red), and DAPI (blue) in the corpus callosum after puerarin treatment in ICH mice. Scale bar = 50 μm. (**H**) Quantification of mature oligodendrocytes (CC1 + Olig2 + cells). In all bar graphs, the overlaid geometric shapes represent individual biological replicates from different experimental groups: circles for the Sham group, triangles for the Sham + Puerarin (Sham + Pu) group, diamonds for the ICH group, and squares for the ICH + Puerarin (ICH + Pu) group. Data are presented as mean ± SEM. Statistical significance was determined by one-way ANOVA followed by Tukey’s post hoc test (* *p* < 0.05, ** *p* < 0.01, **** *p* < 0.0001).

While AKT1 and PARP1 emerged as potential binding targets, the in vivo validation focused on cGAS–STING pathway, the key inflammatory pathway linked to oligodendrocyte injury. To elucidate the mechanisms by which puerarin promoted white matter repair after ICH, we examined the expression of key proteins in the cGAS–STING signaling pathway. Western blot analysis revealed that the protein levels of cGAS and STING were markedly elevated in the corpus callosum of ICH mice compared with sham controls (*p* < 0.01), whereas puerarin treatment significantly reduced their expression (*p* < 0.05;Figure 8A–C). Consistently, phosphorylation of TBK1 was significantly increased after ICH (*p* < 0.01) and was markedly suppressed by puerarin administration (*p* < 0.05; Figure 8A,D). We next assessed the effects of puerarin on oligodendrocyte lineage cell survival. Olig2/TUNEL double immunofluorescence staining suggested that puerarin treatment significantly reduced apoptosis of oligodendrocyte precursor cells (OPCs) in the corpus callosum after ICH (Figure 8E,F). Given that remyelination initiates approximately 14 days after injury and peaks around day 28, we further evaluated myelin repair at day 28 post-ICH (*p* < 0.01). We found that puerarin-treated mice exhibited a significantly reduced demyelinated area compared with vehicle-treated ICH mice (Figure 8G,H). Moreover, the number of CC1^+^ cells, indicative of newly generated mature oligodendrocytes, was markedly increased within lesion regions in puerarin-treated ICH mice (*p* < 0.05).

In summary, these results indicate that puerarin treatment was associated with reduced activation of the cGAS–STING signaling pathway and improved myelin preservation in the context of white matter injury. In parallel, puerarin treatment alleviated blood–brain barrier disruption, collectively contributing to improved neurological outcomes in the ICH model.

## 4. Discussion

ICH is the most severe subtype of stroke, with more than 90% of survivors experiencing persistent neurological dysfunction, including cognitive and motor impairments [21]. However, currently available pharmacotherapies for post-ICH neurological deficits, such as deferoxamine (DFO), tranexamic acid (TXA), and piracetam, have demonstrated limited clinical efficacy [22]. A major limitation of these interventions is that their therapeutic effects are largely confined to the acute phase and fail to address white matter injury (WMI), which critically determines long-term functional recovery. Consequently, the development of effective therapeutic strategies targeting WMI remains a major unmet need in ICH management [23].

cGAS functions as a cytosolic pattern recognition receptor for double-stranded DNA and orchestrates type I interferon–mediated inflammatory responses under pathological conditions. Following ICH, hemoglobin-induced neuronal injury promotes nuclear and mitochondrial DNA leakage into the cytoplasm [24], leading to aberrant activation of the cGAS–STING signaling cascade. Previous studies have shown that this activation occurs predominantly in IBA-1^+^ microglia/macrophages, driving M1 polarization and the release of pro-inflammatory cytokines, such as IL-1β and IL-6, which contribute to oligodendrocyte injury and myelin degeneration [25]. Sustained NF-κB activation downstream of cGAS–STING further amplifies neuroinflammation. In parallel, cGAS–STING signaling has been reported to cross-regulate the EIF2AK2 (PKR) pathway, whereby phosphorylation of eIF2α suppresses the expression of myelin basic protein (MBP) and proteolipid protein (PLP), ultimately impairing oligodendrocyte precursor cell differentiation [26]. Moreover, concomitant disruption of the blood–brain barrier (BBB) facilitates plasma component extravasation into white matter, exacerbating vasogenic edema and demyelination [27]. The cGAS-STING pathway has emerged as a pivotal mediator of sterile neuroinflammation following acute brain injury. Accumulating evidence from both hemorrhagic and ischemic stroke models indicates that damage-associated molecular patterns (DAMPs), such as mitochondrial DNA released from injured cells, can activate the cGAS-STING axis in microglia and astrocytes. This activation triggers a robust type I interferon response and the production of pro-inflammatory cytokines, thereby exacerbating secondary brain damage and functional impairment [28,29]. Collectively, these findings position cGAS–STING as a central regulator of WMI and BBB dysfunction after ICH.

The therapeutic profile of puerarin contrasts with that of canonical, single-target cGAS inhibitors such as RU.521 [30]. While RU.521 is a potent and selective tool compound designed to directly inhibit cGAS catalytic activity [31], puerarin exemplifies the “multi-component, multi-target” mode of action characteristic of many active compounds in traditional Chinese medicine. Beyond its potential interaction with the PARP1/cGAS-STING axis suggested by our data, extensive literature indicates that puerarin can also modulate other pivotal pathways involved in neuroinflammation and oxidative stress, including the NF-κB,JNK/FoxO1, MAPK, and Nrf2 signaling cascades [32,33]. This ability to simultaneously engage multiple therapeutic targets may allow puerarin to exert a more comprehensive and synergistic effect on the intricate pathological network following ICH—addressing not only aberrant DNA sensing but also concomitant inflammatory responses and redox imbalance. Consequently, puerarin may offer a holistic regulatory advantage over highly selective single-pathway inhibitors for promoting neural repair and functional recovery in the complex setting of brain injury.

**Figure 9:**
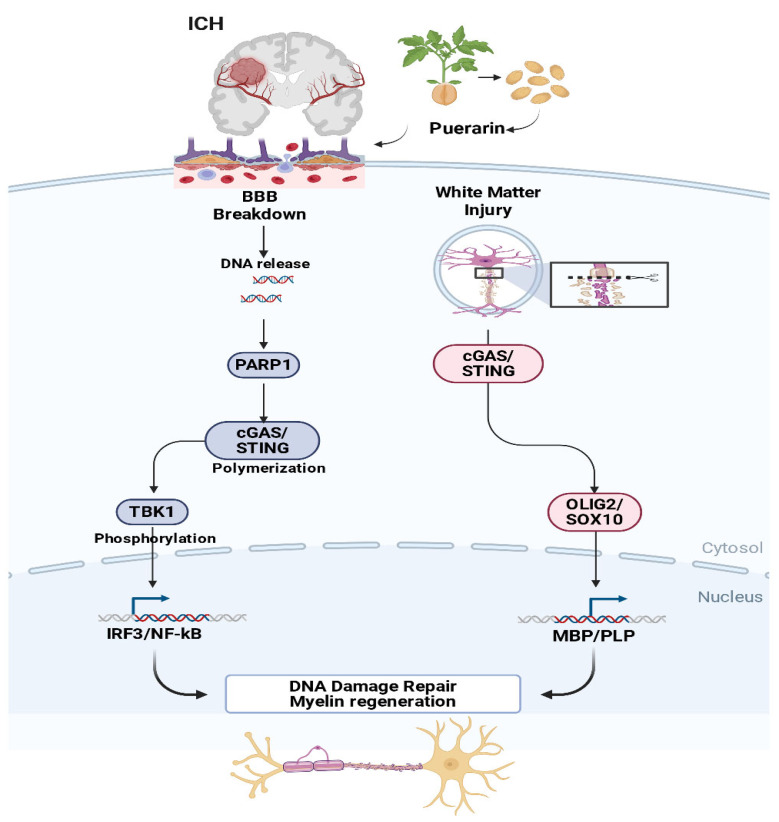
A schematic model illustrating that puerarin may improve DNA damage repair and myelin regeneration after ICH by potentially modulating the cGAS-STING pathway. Puerarin might suppress the activation of this signaling cascade, thereby attenuating the related neuroinflammatory response, which could collectively enhance DNA damage repair and promote myelin regeneration.

In the present study, we systematically evaluated the therapeutic effects of puerarin on WMI and BBB integrity following ICH. Prolonged puerarin administration significantly improved neurological function, particularly at higher doses, accompanied by enhanced cerebral microcirculation and cerebral blood flow. Through an integrative strategy combining network pharmacology, transcriptomic profiling, and machine learning analyses, AKT1 and PARP1 were identified as potential key targets of puerarin. These predictions were further supported by molecular docking and molecular dynamics simulations, which suggested relatively stable interactions between puerarin and AKT1/mTOR, PARP1, and cGAS–STING signaling components. Notably, puerarin exhibited a strong binding affinity toward cGAS, indicating a potential capacity to interfere with cGAS activation. Consistent with these computational findings, puerarin treatment attenuated WMI, restored BBB integrity, reduced oligodendrocyte apoptosis, and may modulated cGAS–STING–related gene expression in vivo. Taken together, our results suggest that Puerarin’s promotion of remyelination may involve, in part, the modulation of the cGAS–STING pathway, thereby providing mechanistic support for targeting this signaling axis in ICH (Figure 9).

The two hub genes identified via network pharmacology analysis were found to function in the development of ICH-associated white matter injury: one acts predominantly as a “disruptor,” promoting injury progression, while the other functions as a “repairer,” facilitating tissue recovery [34]. After ICH, erythrocyte rupture releases heme and iron ions, and damaged neural cells release cytosolic dsDNA. These DAMPs collectively activate the cGAS/STING pathway [35]. Subsequently, the activated cGAS/STING pathway initiates a cascade that enhances the synthesis and secretion of pro-inflammatory mediators, recruits and activates additional inflammatory cells, and forms an “inflammatory storm,” which may attack vulnerable oligodendrocytes and neuronal axons. Furthermore, oxidative stress in oligodendrocytes or their precursor cells induces accumulation of aberrant cytoplasmic DNA, which activates the STING pathway. This activation elicits a signaling cascade leading to type I interferon production and NF-κB–mediated inflammatory responses. The resulting interferons and inflammatory mediators compromise the transcriptional competence of Olig2 and SOX10, ultimately suppressing the transcription of myelin genes (MBP and PLP) [36]. This process further exacerbates white matter damage. Due to its detrimental role, potentially inhibition of the cGAS/STING pathway has been proposed as a therapeutic strategy. Consistent with this, animal studies have confirmed that either pharmacological inhibition of STING or conditional knockout of the Sting gene in microglia effectively attenuates neuroinflammation, brain edema, and motor deficits after ICH [37].

Indeed, beyond the cGAS–STING axis we focused on, several other pathways represent compelling drug targets. Key druggable nodes include: AMPK/PGC1α/Nrf2 axis [38] and P53/ACSL4 axis [39]. Therapeutic strategies targeting these axes, such as DRP1 inhibition, AMPK-SIRT-PGC-1α activation, and reinforcement of mitophagy and BBB integrity by agents like melatonin, puerarin, or schisandrin B, have shown promise in restoring mitochondrial resilience [40]. Therefore, the combination of Puerarin, a GPX4 activator, and a mitochondrial function inhibitor will be tested in future studies.

According to our KEGG and GO enrichment analysis, puerarin was predicted to participate in the negative regulation of the cGAS/STING signaling pathway, positive regulation of PI3K/PKB and TORC1 signaling, fatty acid oxidation, cellular response to nerve growth factor stimulus, and mitochondrial function. Using machine learning methods, AKT1 and PARP1 were consistently ranked highly by both models. Previous literature suggests that cGAS–STING is primarily activated by the release of cytoplasmic DNA [24]. In response to severe DNA damage, PARP1 becomes hyperactivated to repair lesions, often resulting in energy exhaustion and cell death. During cell death or mitosis, compromised nuclear DNA can migrate into the cytoplasm as micronuclei or fragments, while PARP1 activation may also trigger mitochondrial DNA leakage [41]. These aberrant cytoplasmic DNA species are detected by cGAS sensors, triggering the cGAS–STING cascade and subsequent production of type I interferons and inflammatory factors [42]. Notably, AKT1 was predicted to have a potential interaction with PARP1, as well as Olig2 and MBP, through PPI analysis using String, though the precise mechanism remains unclear. In a Parkinson’s disease model, pharmacological activation of AKT1 by compounds such as chlorogenic acid led to phosphorylation and activation of the downstream transcription factor CREB, indirectly suppressing the detrimental activity of PARP1 and inhibiting the cell death pathway [43,44]. It is hypothesized that after ICH, overactivated PARP1 generates excessive PAR polymers that serve as critical messengers for death signaling by activating the cGAS–STING pathway [35]. Phosphorylated AKT1 may then activate CREB, which binds to the RNF146 promoter and enhances RNF146 protein expression. RNF146 may sequester PAR polymers, blocking PARP1-driven programmed cell death and ultimately attenuating white matter injury [45].

Recurrent optogenetic stimulation of demyelinated axons in the motor cortex has been shown to promote remyelination and restore conduction. Similarly, activation of glutamatergic neurons in the medial prefrontal cortex enhances OPC differentiation in the corpus callosum, improving myelin repair and cognitive function [46]. GO analysis suggested that puerarin targets may be involved in excitatory postsynaptic potential, which could facilitate OPC proliferation, migration, and differentiation through glutamate receptor activation [47]. However, these invasive techniques have not yet been applied clinically, and suitable pharmacological agents for promoting myelination remain limited.

Furthermore, therapies targeting the pathological microenvironment, particularly neuroinflammation, have been reported to enhance remyelination [48]. Puerarin has been shown to potentially inhibit apoptosis and reduce inflammation. Targeting the cGAS-STING axis has yielded a spectrum of outcomes that inform our rationale. In neuroinflammatory contexts, dimethyl fumarate downregulated cGAS-STING-mediated cytokines in ALS models [49], while tetrahydroxy stilbene glycoside—a botanical derivative from Polygonum multiflorum—reduced microglial M1 polarization in Alzheimer’s disease via STING inhibition [50], supporting CNS applicability of natural compounds. However, several failed or limited attempts underscore key barriers. DMXAA (ASA404) failed clinically due to species specificity and systemic IFN-β therapy, despite targeting the same IRF3-IFN-β axis, remains severely dose-limited by neurotoxicities, cognitive dysfunction and depression [51]. Additionally, STING-activating nanoparticles face BBB permeability challenges and cytokine-release risks, restricting utility in acute neurological insults like ICH. Against this backdrop, Puerarin emerges as a multi-modal STING modulator with documented oral bioavailability, favorable safety margins, and pleiotropic anti-inflammatory effects that may circumvent the narrow therapeutic windows and species-specificity issues plaguing synthetic agents. In our study, puerarin effectively mitigated inflammation and concurrently ameliorated myelin loss through a mechanism partially mediated by cGAS-STING signaling. Although the precise anti-inflammatory mechanisms in demyelinating diseases remain unclear, this warrants further investigation. Its dual role in promoting remyelination and suppressing inflammation highlights its therapeutic potential for multiple sclerosis and other white matter lesions [12].

Despite its therapeutic potential, the long-term use of puerarin is associated with several notable drawbacks that warrant careful consideration. Firstly, toxicological studies indicate potential reproductive toxicity, as puerarin has been shown to induce apoptosis in mouse embryonic cells, disrupt embryo implantation in rats, and reduce sperm activity. Evidence of hepatotoxicity also exists, with in vitro and in vivo studies reporting the upregulation of pro-apoptotic markers like Bax in liver cells. Clinically, the injectable form of puerarin has been linked to adverse reactions involving multiple systems, including immune, digestive, and cardiovascular systems, with severe cases even reporting fatalities. These reactions occur most frequently in elderly patients, predominantly within 48 h of administration. Furthermore, the inherent poor pharmacokinetic profile of puerarin—characterized by low solubility, poor oral bioavailability and may necessitate high or frequent dosing, potentially exacerbating toxicity risks. While some studies suggest a relatively safe profile at lower doses, these identified risks highlight the necessity for stringent dose optimization, vigilant clinical monitoring, and the development of advanced drug delivery systems to improve its safety and brain-targeting efficiency for ICH.

Although the collagenase IV–induced ICH model is highly standardized, its acute and mono-factorial pathology differs from the multifactorial and chronic progression of human ICH, limiting clinical translatability. Moreover, key pharmacokinetic parameters of puerarin—including systemic bioavailability, BBB penetration, and brain tissue target occupancy—have not been fully characterized. Due to their high metabolic demands and limited repair capacity, oligodendrocytes are particularly susceptible to oxidative stress [52], making ICH-induced damage more severe in white matter tracts dominated by oligodendrocytes.

Some potential limitations deserve special attention in our study. Direct molecular evidence confirming binding between puerarin and its targets is still lacking. Further experimental validation, such as molecular pull-down assays, enzymatic activity measurements, and quantitative assessment of proteins associated with OPC differentiation and myelin debris clearance, is needed in both cellular and animal models. Additionally, given that puerarin was administered before ICH induction, our findings primarily reflect preventive or mechanistic effects rather than direct therapeutic efficacy. Future studies using post-ICH treatment protocols are warranted for translational validation. Another consideration is that the translational potential of these findings necessitates careful consideration of the dosage used in this study. While the high dose used (200 mg/kg) translates to a human-equivalent dose at the upper limit of reported clinical ranges (1 g/day), future pharmacokinetic and dose-optimization studies are essential to define the therapeutic window for post-ICH treatment. Furthermore, while our data strongly associate puerarin with cGAS-STING suppression and functional improvement, a definitive causal link remains to be established. Future studies employing genetic or pharmacological tools to directly manipulate this pathway are crucial to confirm that the observed benefits are specifically mediated through cGAS-STING inhibition. Finally, the functional assessment was conducted up to 14 days post-ICH. Longer-term behavioral and histological evaluations are needed to determine the durability of puerarin’s neuroprotective effects and its impact on chronic recovery processes.

Future studies should employ CRISPR/Cas9 to generate STING conditional knockout models to validate the role of this pathway. Hypertensive ICH models could better recapitulate clinical pathophysiology. Extending the therapeutic window to the subacute phase should be investigated to evaluate puerarin’s effects on white matter repair. Systematic pharmacokinetic studies in ICH models are also essential to assess brain distribution, metabolism, and optimal dosing, enhancing translational potential.

## 5. Conclusions

In conclusion, our data indicate that puerarin may protect against neurological damage after ICH, at least in part by mitigating white matter injury and repairing the blood–brain barrier. It may achieve this by reducing oligodendrocyte apoptosis, promoting remyelination, and improving cerebral microcirculation. Mechanistically, puerarin may modulate key signaling pathways, including cGAS/STING, AKT1, and PARP1, thereby stabilizing cellular homeostasis and mitigating inflammatory damage. Integrating bioinformatic predictions with in vivo validation, our findings highlight puerarin as a promising therapeutic agent for enhancing white matter repair and functional recovery following ICH. Future investigations could focus on confirming these molecular mechanisms in advanced ICH models, optimizing dosing regimens, and evaluating pharmacokinetics and brain penetration to accelerate translational application.

## Figures and Tables

**Table 1:** Molecular docking energy.

Gene	Affinity Score (kcal/mol)	PDB ID
AKT1	−8.5	3OCB
PI3K	−9.1	4L23
MTOR	−9.2	1NSG
PARP1	−8.1	6NRH
STING	−8.9	4EMT
cGAS	−10.0	9MDC

Molecular docking was performed between puerarin and six core targets from the two signaling pathways, and the binding energies between puerarin and these potential pathway targets were presented.

## Data Availability

The original contributions presented in this study are included in the article: Further inquiries can be directed to the corresponding authors.

## Supplementary Materials

The following supporting information can be downloaded at: https://www.mdpi.com/article/10.3390/biology15030277/s1, Figure S1: Molecular dynamics simulation of AKT1 and PARP1; Figure S2: Assessment of BBB permeability; Figure S3: The original western blot images for Figure 2; Figure S4: The original western blot images for Figure 8.

## Abbreviations

The following abbreviations are used in this manuscript:

ICH	Intracerebral hemorrhage
WMI	White matter injury
BBB	Blood–brain barrier
CNS	Central nervous system
TOM	Topological overlap matrix
WGCNA	Weighted Gene Co-expression Network Analysis
DEGs	Differentially expressed genes
TEM	Transmission electron microscopy
