# Supplementary material for: Puerarin Attenuates White Matter Injury and Blood–Brain Barrier Disruption After Intracerebral Hemorrhagic Stroke via cGAS-STING Axis
Source: Biology (Basel). 2026 Feb 3;15(3):277. doi: 10.3390/biology15030277 (PMC12897197; doi:10.3390/biology15030277)
Supplement: Supplementary file 1 [file biology-15-00277-s001.zip › Full Western blot.pdf]

Western blot figures

Figure S3: The original western blot images for Figure 2.

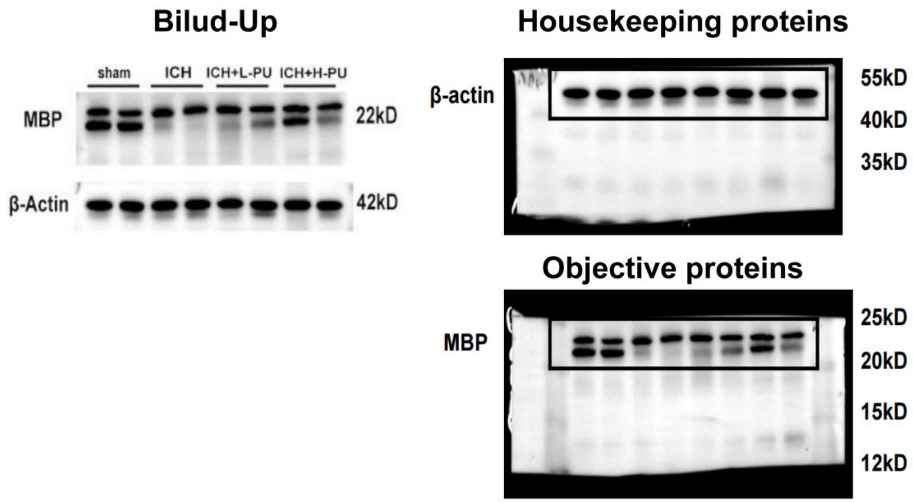

Western blot membrane of MBP (~22 kDa) protein detected with rabbit anti-MBP (1:2000, 10458-1-AP, Proteintech) antibody and mouse anti-Beta Actin (1:5000, 66009-1-Ig, Proteintech). Gel-separated proteins were transferred to PVDF membranes (0.22 μm pore size; Millipore, Billerica, MA, USA) using wet transfer at 200mA for 1 hours at 4°C. Membranes, incubated with a HRP-conjugated Goat Anti-Rabbit IgG(H+L) (SA00001-2; 1:5000; Proteintech), HRP-conjugated Goat Anti-Mouse IgG(H+L) (SA00001-1; 1:5000; Proteintech)were developed with SuperSignal™ West Pico PLUS Kit (34580，Thermo Fisher Scientific). Weight marker (molecular weight in kDa): Thermo Scientific™/PageRuler™ Prestained Protein Ladder, 10 to 180 kDa; catalogue number: 26616. Blot images, prior to the densitometry readings, were converted to grayscale with ImageJ (ImageJ v.1.8.0, National Institutes of Health, Maryland, USA).

Figure S4: The original western blot images for Figure 8.

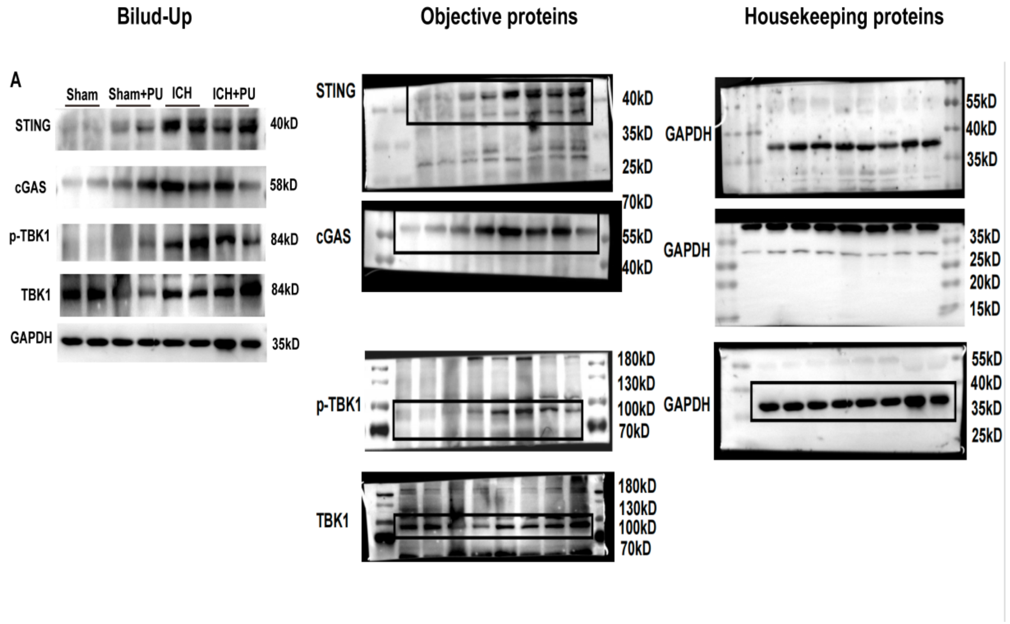

Western blot membrane of STING (~40 kDa) protein，cGAS(~58kDa) protein, p-TBK1(~84kDa) protein,TBK1 (~84kDa) protein, GAPDH (~35kDa) were respectively proteindetected with rabbit anti-STING (1:1000, 19851-1-AP, Proteintech) antibody, rabbit anti-cGAS (1:2000, PQA3430, Abmart), rabbit anti-p-TBK1 (1:1000, 5483, Cell Signaling
Technology), rabbit anti-TBK1 (1:1000, 3504, Cell Signaling Technology) and rabbit anti-GAPDH(1:5000, 5174, Abcam). Gel-separated proteins were transferred to PVDF membranes (0.22 µm pore size; Millipore, Billerica, MA, USA) using wet transfer at 100V for 1.5 hours at 4°C. Membranes, incubated with a HRP-conjugated Goat Anti-Rabbit IgG(H+L) (SA00001-2; 1:5000; Proteintech), were developed with SuperSignal™ West Pico PLUS Kit (34580, Thermo Fisher Scientific). Weight marker (molecular weight in kDa): Thermo Scientific™/PageRuler™ Prestained Protein Ladder, 10 to 180 kDa; catalogue number: 26616. Blot images, prior to the densitometry readings, were converted to grayscale with ImageJ (ImageJ v.1.8.0, National Institutes of Health, Maryland, USA).

The densitometry readings/intensity ratio of each band

Sample ID	Objective protein intensity	Housekeeping protein intensity	Intensity ratio (Objective protein/Housekeeping protein)	The corresponding bands /picture number
Sham1	28295.59	26012.42	1.087772303	Figure2D,Lane 1-MBP
Sham2	25944.52	26068.47	0.995245214	Figure2D,Lane 2-MBP
ICH1	18029.23	27676.76	0.651421265	Figure2D,Lane 3-MBP
ICH2	15801.23	28609.59	0.552305363	Figure2D,Lane 4-MBP
ICH+L-PU1	18954.05	24954.05	0.759558068	Figure2D,Lane 5-MBP
ICH+L-PU2	20120.12	29834.12	0.674399647	Figure2D,Lane 6-MBP
ICH+H-PU1	25911.25	26288.64	0.98564437	Figure2D,Lane 7-MBP
ICH+H-PU2	19056.64	25195.95	0.756337427	Figure8D,Lane 8-MBP
Sham1	7422	17035.25	0.43568483	Figure8D,Lane 1-STING
Sham2	5381.42	27837.12	0.193318131	Figure8D,Lane 2-STING
Sham+PU1	11513.64	31808.73	0.361964781	Figure8D,Lane 3-STING
Sham+PU2	13598.47	28577.54	0.475844667	Figure8D,Lane 4-STING
ICH1	25884.83	26590.59	0.97345828	Figure8D,Lane 5-STING
ICH2	26094.3	22677.54	1.150667136	Figure8D,Lane 6-STING
ICH+PU1	19824.71	28748.88	0.689581994	Figure8D,Lane 7-STING
ICH+PU2	24875.54	27195.9	0.914679786	Figure8D,Lane 8-STING
Sham1	5153.28	22845.1	0.22557485	Figure8D,Lane 1-cGAS
Sham2	9312.81	23236.35	0.400786268	Figure8D,Lane 2-cGAS
Sham+PU1	10360.93	22925.62	0.451936742	Figure8D,Lane 3-cGAS
Sham+PU2	16820.35	23455.64	0.71711324	Figure8D,Lane 4-cGAS
ICH1	25980.37	25398.71	1.022901163	Figure8D,Lane 5-cGAS
ICH2	20089.59	26730.59	0.751558046	Figure8D,Lane 6-cGAS
ICH+PU1	20442.93	23847.28	0.857243677	Figure8D,Lane 7-cGAS
ICH+PU2	10842.88	20545	0.527762473	Figure8D,Lane 8-cGAS
Sham1	6380.31	17684.35	0.360788494	Figure8D,Lane 1-p-TBK1/TBK1
Sham2	3926.13	20817.3	0.188599386	Figure8D,Lane 2-p-TBK1/TBK1
Sham+PU1	3188.62	20366.12	0.156564923	Figure8D,Lane 3-p-TBK1/TBK1
Sham+PU2	7136.67	14618.35	0.488199421	Figure8D,Lane 4-p-TBK1/TBK1
ICH1	14891.2	20619.25	0.722198916	Figure8D,Lane 5-p-TBK1/TBK1
ICH2	19019.57	19174.64	0.991912756	Figure8D,Lane 6-p-TBK1/TBK1
ICH+PU1	13882.81	15781.71	0.879677171	Figure8D,Lane 7-p-TBK1/TBK1
ICH+PU2	8425.45	19295.35	0.436657018	Figure8D,Lane 8-p-TBK1/TBK1
