# Supplementary material for: Puerarin Attenuates White Matter Injury and Blood–Brain Barrier Disruption After Intracerebral Hemorrhagic Stroke via cGAS-STING Axis
Source: Biology (Basel). 2026 Feb 3;15(3):277. doi: 10.3390/biology15030277 (PMC12897197; doi:10.3390/biology15030277)
Supplement: Supplementary file 1 [file biology-15-00277-s001.zip › Supplementary Figures.pdf]

Supplementary Figures

Supplementary Figure S1 Molecular dynamics simulation of AKT1 and PARP1

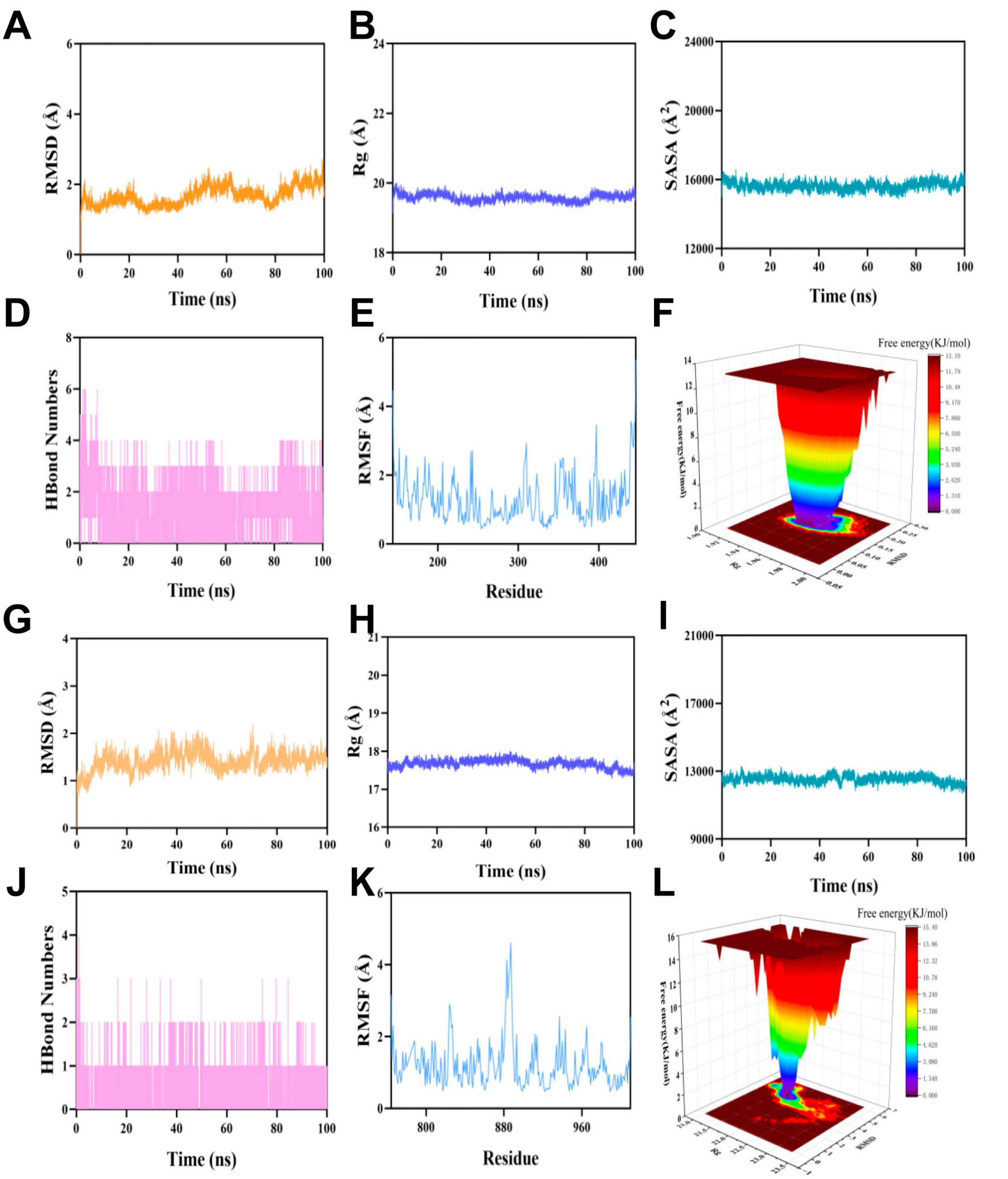

(A-F) Molecular dynamics simulation of the AKT1-Puerarin complex. (G-L) Molecular dynamics simulation of the PARP1-Puerarin complex. RMSD, root mean square deviation. RMSF, root mean square fluctuation. Rg, Radius of gyration. H-bonds, hydrogen bonds. SASA, solvent-accessible surface area. FEL, free energy landscape.

Supplementary Figure S2-Assessment of BBB permeability

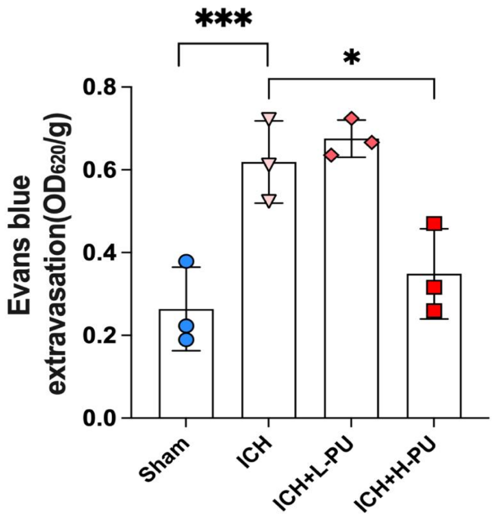

Quantification of Evans Blue extravasation in the ipsilateral hemisphere of ICH mice, indicating blood-brain barrier permeability following treatment. Evans blue extravasation was quantified by measuring absorbance at 620 nm and normalized to tissue weight (OD620/g). Data are presented as the mean ± SEM (n = 3 mice per group). Statistical significance was determined by one-way ANOVA followed by Tukey's post hoc test, with ***p < 0.001 and *p < 0.05.
